# HIV-1 Superinfection in the Antiretroviral Therapy Era: Are Seroconcordant Sexual Partners at Risk?

**DOI:** 10.1371/journal.pone.0005690

**Published:** 2009-05-28

**Authors:** Mary S. Campbell, Geoffrey S. Gottlieb, Stephen E. Hawes, David C. Nickle, Kim G. Wong, Wenjie Deng, Thomas M. Lampinen, Nancy B. Kiviat, James I. Mullins

**Affiliations:** 1 Department of Medicine, School of Medicine, University of Washington, Seattle, Washington, United States of America; 2 Department of Microbiology, University of Washington, Seattle, Washington, United States of America; 3 Department of Epidemiology, School of Public Health & Community Medicine, University of Washington, Seattle, Washington, United States of America; 4 Rosetta InPharmatics/Merck & Co., Seattle, Washington, United States of America; 5 Department of Pathology, School of Medicine, University of Washington, Seattle, Washington, United States of America; 6 School of Population and Public Health, University of British Columbia, Vancouver, Canada; Comprehensive AIDS Reseach Center, China

## Abstract

**Background:**

Acquisition of more than one strain of human immunodeficiency virus type 1 (HIV-1) has been reported to occur both during and after primary infection, but the risks and repercussions of dual and superinfection are incompletely understood. In this study, we evaluated a longitudinal cohort of chronically HIV-infected men who were sexual partners to determine if individuals acquired their partners' viral strains.

**Methodology:**

Our cohort of HIV-positive men consisted of 8 couples that identified themselves as long-term sexual partners. Viral sequences were isolated from each subject and analyzed using phylogenetic methods. In addition, strain-specific PCR allowed us to search for partners' viruses present at low levels. Finally, we used computational algorithms to evaluate for recombination between partners' viral strains.

**Principal Findings/Conclusions:**

All couples had at least one factor associated with increased risk for acquisition of new HIV strains during the study, including detectable plasma viral load, sexually transmitted infections, and unprotected sex. One subject was dually HIV-1 infected, but neither strain corresponded to that of his partner. Three couples' sequences formed monophyletic clusters at the entry visit, with phylogenetic analysis suggesting that one member of the couple had acquired an HIV strain from his identified partner or that both had acquired it from the same source outside their partnership. The 5 remaining couples initially displayed no evidence of dual infection, using phylogenetic analysis and strain-specific PCR. However, in 1 of these couples, further analysis revealed recombinant viral strains with segments of viral genomes in one subject that may have derived from the enrolled partner. Thus, chronically HIV-1 infected individuals may become superinfected with additional HIV strains from their seroconcordant sexual partners. In some cases, HIV-1 superinfection may become apparent when recombinant viral strains are detected.

## Introduction

Data from case reports and cohort studies have established that infection with more than one strain of human immunodeficiency virus type 1 (HIV-1)– termed dual infection– does occur. Dual infection encompasses both co-infection, acquisition of two separate viral strains during primary infection, and superinfection, acquisition of one or more viral strains after seroconversion. The phenomena of HIV-1 dual and superinfection have been reported in a variety of risk groups, including men who have sex with men (MSM), heterosexual women, infants, and injection drug users (IDU) [1]–[23], and are relevant to HIV clinical care, epidemiology, and the design of prevention strategies. Superinfection may lead to acquisition of drug-resistant strains [24], [25] and has been associated with accelerated disease progression [10], [26]. The presence of multiple viral strains within a host permits inter- and intra-subtype recombination, which increases global HIV diversification [5], [21], [22], [27]–[29]. Finally, research on dual and superinfection is pertinent to the development of preventive approaches, as it is clear that in some instances HIV-positive individuals are vulnerable to repeated infection with other viral strains.

A subgroup for which limited data on dual infection exists is that of HIV-1 seroconcordant partners. Few such cohorts are available for evaluation, yet insight into the risks of sexual transmission in seroconcordant couples is highly desirable for the purposes of individual and public health. Studying sexual partners also allows for greater scrutiny for evidence of viral transmission than in other individuals, using both molecular and computational techniques. With this rationale, we undertook the following examination for dual and superinfection in our cohort of HIV-1 seroconcordant long-term sexual partners who were MSM.

## Materials and Methods

### Participants

Sixteen HIV-1-infected MSM who identified themselves as long-term sexual partners in the Male Anal Health Study (MAHS) were included in this analysis. The MAHS is a longitudinal cohort designed to examine the role of HIV-1 in the development of anal dysplasia [30], composed of 337 men, recruited in Seattle, Washington between 1996–2000. Volunteers gave written informed consent for participation. The study was approved by the University of Washington Human Subjects Review Committee and was conducted in accordance with the principles of the Helsinki Declaration. Participants returned at 4-month intervals and completed questionnaires regarding demographics, behavior, and health and underwent physical examinations and collection of blood and anorectal swabs. In this paper, participants were designated with identification numbers different from those used during study follow-up to protect the confidentiality of the volunteers.

### Specimen collection and clinical testing

At each visit, serum, whole blood, and anorectal swabs were obtained for HIV-1 quantification, as described [30]. Specimens available for this study included DNA from peripheral blood mononuclear cells (PBMC) and anorectal swabs.

### HIV-virus isolation, PCR, cloning, and sequencing

We examined HIV-1 sequences from PBMC from the initial and final study visits. In some individuals, we were also able to obtain HIV-1 sequences from cell pellets from anorectal swab specimens. Our methods for DNA extraction, PCR, cloning, and sequencing are described in detail in previous publications [31]–[34]. Briefly, we used endpoint serially diluted genomic DNA extracted from PBMC or anorectal swabs to perform nested PCR to amplify the ∼637 nucleotide-long C2-V5 fragment of the HIV-1 envelope gene (*env*). PCR products were cloned in plasmids and sequenced with the dideoxy terminator technique. To minimize risk of sample mix-up and contamination, we avoided working with specimens from a given subject and his partner simultaneously. Sequences were submitted to GenBank (accession numbers FJ975207–FJ975545).

### Phylogenetic analysis

We screened for specimen mix-up and contamination using the Los Alamos National Laboratory (LANL) HIV sequence database (http://hiv-web.lanl.gov) [35] and our internal sequence database (http://indra.mullins.microbiol.washington.edu/blast/viroblast.php). Alignments were created with CLUSTALW [36] and manually adjusted in MacClade v4.08 [37]. PAUP* v4.0b10 [38] was used to generate distance matrices and neighbor-joining (NJ) and maximum-likelihood (ML) phylogenies.

### Strain-specific PCR

For couples whose HIV-1 sequences were phylogenetically distinct, we sought to identify low-level superinfection using specific primers to amplify a subject's viral variants within his partner's PBMC-derived HIV DNA, the only blood-derived specimens available for this study. We designed primers using nucleotide alignments that flanked unique sequence regions in each subject's *env* gene. Our goal was to target regions of *env* that were conserved in an individual participant, but distinct from that in the partner and thus could best distinguish viruses in the couple. Nested PCR was performed, using primers DR7 and DR8 [31] in the first round, which amplifies the 637 nucleotide C2-V5 region of envelope, and specific primers for the second round. Gel electrophoresis was used to ascertain whether fragments of appropriate length had been amplified. We first tested DNA from *env* clones from each subject and his partner to determine the sensitivity and specificity of the primers, with a limit of detection of 1–2 copies per PCR reaction. After it was found that the primers both amplified the region of interest from a subject's viral clone and failed to generate non-specific bands from that of his partner, PCR was performed using PBMC-derived DNA.

### Recombination analysis

To further investigate for superinfection, we searched for viral recombination in pairs whose viruses were phylogenetically distinct (pairs A, B, E, F, and I) using two methods. We first used the Recombinant Identification Program (RIP version 3.0, http://www.hiv.lanl.gov/content/sequence/RIP/RIP.html). The algorithm uses a sliding window method to determine if a query sequence from an individual matches sequences in a background alignment of viral sequences derived from that individual and his partner. We performed this analysis for pairs A, B, E, F, and I, using a window size of 100 and significance threshold of 90%.

Since the various recombination detection methods that are available may yield different results, we also evaluated the data with the InSites algorithm (http://indra.mullins.microbiol.washington.edu/cgi-bin/InSites/index.cgi), which has been used to detect recombinant sequences by visual inspection of shared nucleotide sites in an alignment [39]. We used CLUSTALW and MacClade v4.08 to create separate nucleotide alignments for each pair, which in turn allowed us to derive a consensus sequence for the pair. Using the consensus sequence as the reference sequence, we used the alignment as input for the InSites program, which generates an output of phylogenetically informative sites, i.e. sites in the alignment with nucleotides that differ from the reference sequence, but are shared by two or more non-reference sequences, as described in Gottlieb *et al.* [39]. This permitted visualization of sites with nucleotides that were present in a large portion of one subject's sequences that were also shared by a subset of another subject's sequences. We performed this analysis with sequences from enrolled couples, as well as with sequences from pairs of subjects in the cohort that had no known epidemiologic linkage.

## Results

### Demographic and clinical data

We evaluated HIV-1 sequences in 8 pairs of MSM at two visits 11–71 months apart (median 55.3 months). At study entry, their CDC-defined HIV disease classification ranged from A1-C3. Five had baseline CD4^+^ counts ≤200 cells/ul and eleven had detectable plasma viral loads (Table 1).

**Table 1:** Phylogenetic and recombination data in clinical and demographic context.

Couple	Subject	CD4^+^ cells/uL	HIV RNA copies/mL	Year Partnership Began	Year Perceived HIV Infection	Date HIV+	Visits with ART		Median HIV RNA in follow-up (Range)	Visits with detectable HIV RNA		Follow-up period (mo)	Phylogenetic linkage present	Recombinants
							No.	%		No.	%			
A	1	119	75208	1989	1989	Jan-1989	15/15	100	21633 (124–121667)	15/15	100	71.2	No (Subject 1 - dual infection)	No
	2	471	32214		1995	Jan-1995	7/13	54	4783 (<50–1425238)	10/13	77	67.5		No
B	3	486	189729	Not reported	1985	May-1989	12/14	86	<50 (<50–385797)	2/14	14	69.7	No	No
	4	311	<50		1982	Jun-1987	12/12	100	1029 (<50–1733)	10/13	77	66.1		Yes*
D	5	36	<50	1992	1988	Dec-1988	11/11	100	<50	0/11	0	41.2	Yes	-
	6	138	4309		1988	Feb-1998	8/10	80	514 (<50–174212)	6/10	60	36.1		-
E	7	763	<50	1993	1981	Nov-1987	0/11	0	<50	0/11	0	65	No	No
	8	729	61025		1996	Aug-1996	4/9	44	1260 (<50–61025)	8/9	89	53.7		No
F	9	80	250	1982	1982	Mar-1990	12/12	100	11633 (<50–88431)	12/13	92	59.1	No	Yes
	10	80	127053		1982	May-1990	10/11	91	<50 (<50–354454)	7/14	50	59.1		No
G	11	623	257899	1992	1995	Jul-1997	9/10	90	<50 (<50–257899)	1/10	10	38.7	Yes	-
	12	439	53705		1995	Jul-1997	10/11	91	<50 (<50–53705)	2/13	15	56.8		-
H	13	431	192	1987	1997	Apr-1997	4/4	100	740 (192–7821)	4/4	100	11.3	Yes	-
	14	505	<50		1996	Apr-1997	7/7	100	<50	0/7	0	48.5		-
I	15	462	<50	1993	1986	Jan-1988	8/8	100	<50	0/8	0	41.9	No	No
	16	922	8544		1991	Jul-1991	7/8	88	<50 (<50–14271)	6/8	75	41.9		No

Clinical and demographic data. Recombinants column signifies recombination detected with RIP 3.0.

* significant at 90% confidence level (z-test).

These couples reported sexual partnerships of 3 to 15 years duration prior to enrollment in the MAHS. In some cases, the subjects' perception of the timing of their HIV acquisition pre-dated partnership formation, but others reported a perceived time of HIV infection and laboratory confirmation which corresponded with partnership formation (Table 1).

All but one subject underwent treatment with ART while enrolled. However, only 4 had sustained virological suppression at all visits (Table 1). On a per-couple basis, it was evident that all couples had periods during which at least one partner had a detectable viral load, ranging from 10–100% of visits.

Although no infections with gonorrhea, chlamydia, or syphilis were reported during the study, all participants had anogenital HPV lesions and participants from 5 couples (6 individuals) reported anogenital herpes simplex virus (HSV) recurrences (Table 2). None of the subjects developed AIDS-defining conditions or CD4^+^ counts ≤200 cells/ul during the study.

**Table 2:** Anogenital lesions during follow-up.

Couple	Subject	Anal warts	Penile warts	Anogenital HSV	Episodes/couple
A	1	4	-	-	7
	2	1	1	1	
B	3	-	-	3	5
	4	2	-	-	
D	5	-	-	-	9
	6	2	-	7	
E	7	-	-	4	4
	8	-	-	-	
F	9	6	9	5	24
	10	2	-	2	
G	11	-	2	-	2
	12	-	-	-	
H	13	-	-	-	1
	14	-	1	-	
I	15	1	-	-	1
	16	-	-	-	

Number of visits with anogenital lesions due to human papillomavirus and herpes simplex virus reported during follow-up.

### Behavioral data

Surveys of self-reported sexual behavior were available for all but one participant at study entry and during follow-up (Table 3). At baseline, 9 subjects reported having casual male partners in addition to their primary partner within the past year, with a median of 1.5 partners, range 1–50 (Table 3). There were a median of 4.5 (range 0–40) and 6 (range 0–40) episodes of insertive and receptive anal intercourse respectively during the 4 months prior to study entry, ∼77% of which took place without condoms.

During the follow-up period, representing ∼68 person-years of observation, only 3 subjects reported monogamous relationships with their enrolled partners. The median number of additional sexual partners was 10.5 (range 0–24). Participants reported 2091 episodes of anal intercourse during the follow-up period, ∼73% of which were unprotected. On a per-couple basis, the proportion of episodes of unprotected anal intercourse ranged from 0 to 98%. The behavioral survey did not include queries about the frequency with which subjects had intercourse with each of their partners, so the risk of acquisition of additional HIV strains from the partner under study relative to other casual partners could not be quantified.

**Table 3:** Pooled behavioral data at study entry and during follow-up.

	Baseline Behavioral Data						Follow-up Behavioral Data				
	Partners past yr	Partners past 4 mo	Insertive without condom	Receptive without condom	Total Insertive	Total Receptive	Partners outside primary relationship	Insertive without condom	Receptive without condom	Total Insertive	Total Receptive
Total	109	27	119	105	147	143	167	905	629	1249	842
%	-	-	81%	73%	-	-	-	72%	75%	-	-
Median/subject	1.5	1	3	4	6	5	10.5	49	27	80	29

Pooled behavioral data during follow-up.

### Sequence and phylogenetic data

**Figure 1:**
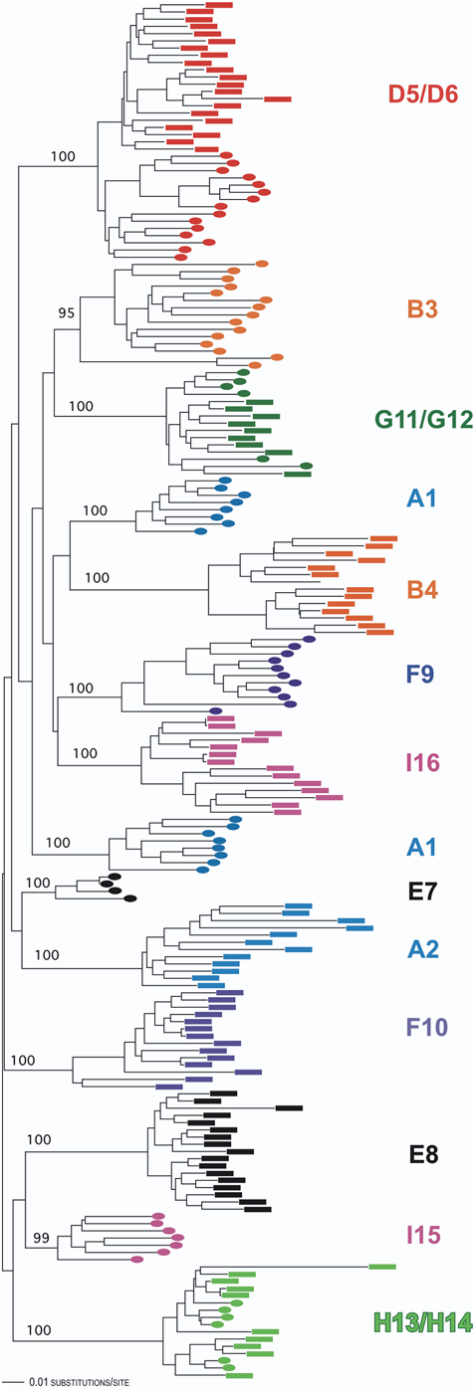
Neighbor-joining phylogenetic tree showing PBMC-derived C2-V5 HIV envelope gene sequences from all members of the cohort with bootstrap values calculated from 1000 bootstrap replicates for each major branch. Couples are designated by colored letters (A, B, and D–I) and correspond to the couples in Table 1. Colored bars and ovals distinguish sequences from partners in each couple.

During the first phase of sequencing, we obtained 206 independent *env* sequences (C2-V5 region) from endpoint dilution PCR reactions, which amplified individual viral templates, (median 12.5 per subject, range 4–21). Three of the 8 couples' HIV *env* sequences clustered in monophyletic groups, (couples D, G, and H, Figure 1), suggesting that one partner may have acquired HIV from the other, that they both acquired HIV from the same individual or other closely-linked individuals, or that following superinfection the second strain displaced the strain acquired during primary infection. No monophyletic lineages were detected in sequences from the remaining 5 couples (couples A, B, E, F, and I), thus initially suggesting absence of transmission or superinfection.

**Figure 2:**
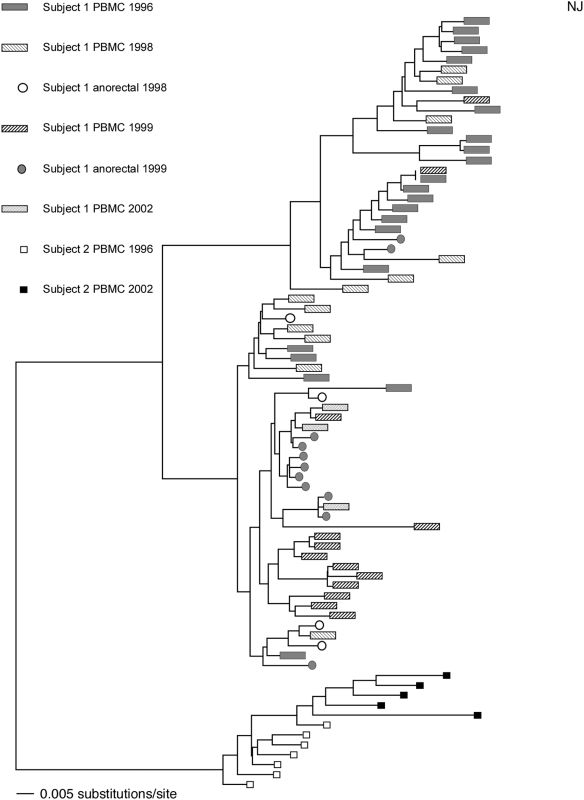
Neighbor-joining tree showing PBMC- and anorectal mucosa-derived sequences from subject 1 and PBMC-derived sequences from subject 2. Subject 1's sequences are clustered on two separate branches, signifying infection with two different HIV strains, both of which are distinct from his partner's isolates (subject 2).

**Figure 3:**
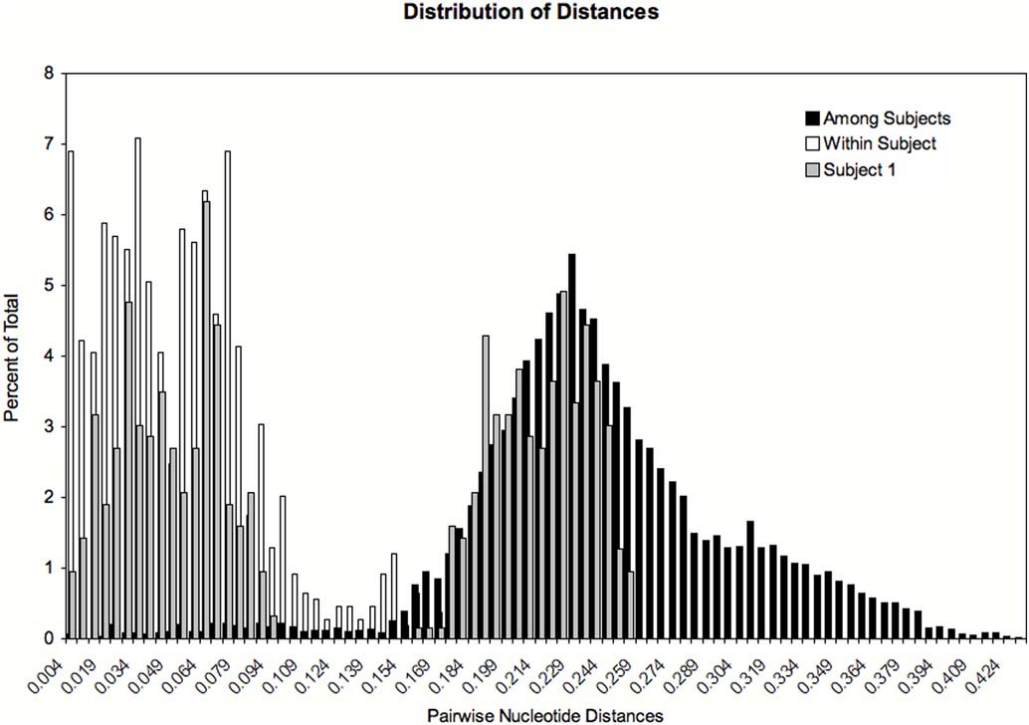
Histogram showing the distribution of pairwise nucleotide distances within subject 1 versus those within and between the remaining subjects in the cohort. Distances were calculated using an HKY85+I+Γ model estimated via maximum likelihood. The histogram contains two peaks for subject 1, which overlie the peaks with distances for ‘among subjects’ and ‘within subjects’ for the cohort, demonstrating the dual HIV infection in subject 1, with his two distinct sets of viral isolates.

Subject A1 had sequences from the first and last study visits that contained two distinct lineages indicating dual infection, but neither strain clustered with those from his partner, subject A2 (Figure 2). Figure 3 shows the distances for subject A1's sequences superimposed upon the distribution of pairwise nucleotide distances within and between patients in the remainder of the cohort, revealing a bimodal distribution, with distances comparable to distances within and between other subjects in the cohort. Finally, additional analyses confirmed that subject A1's viral sequences fell on two different branches when a phylogenetic tree was constructed using sequences from epidemiologically unlinked HIV-1 subtype B-infected subjects from major cities in the United States as described in Anderson et al. 2003 (data not shown) [40], also supporting the presence of dual infection.

We undertook a second phase of sequencing for the 5 pairs whose viruses were phylogenetically unlinked. We obtained sequences from anorectal swabs in subjects A1 (N = 15), A2 (N = 35), B3 (N = 15), B4 (N = 25), and F10 (N = 18) and from PBMC at 2 additional time points in subject A1 (N = 36) and 3 additional time points in subject F9 (N = 4). Amplification of viral DNA from cell pellets from anorectal swab specimens in couples E and I was unsuccessful. This second phase of sequencing did not reveal phylogenetic linkage between the partners in any of these pairs, again suggesting that superinfection was not occurring.

### Strain-specific PCR results

Strain-specific PCR was performed on the PBMC DNA specimens from Pairs A, B, and F. Primers were able to detect each subject's specific variants at a sensitivity of 1–2 copies per reaction and no non-specific amplification of partner's DNA was seen (data not shown). The amount of DNA tested ranged from 0.5 to 2.7 ug, estimated to contain 8–236 viral templates (the product of the number of PBMC and the proviral copy number in each specimen). In Pairs E and I, PCR reactions with specific primers were unsuccessful due to insufficient specificity. The strain-specific PCR did not show evidence of the enrolled partners' viral variants in any of the subjects tested.

### Recombination analysis

**Figure 4:**
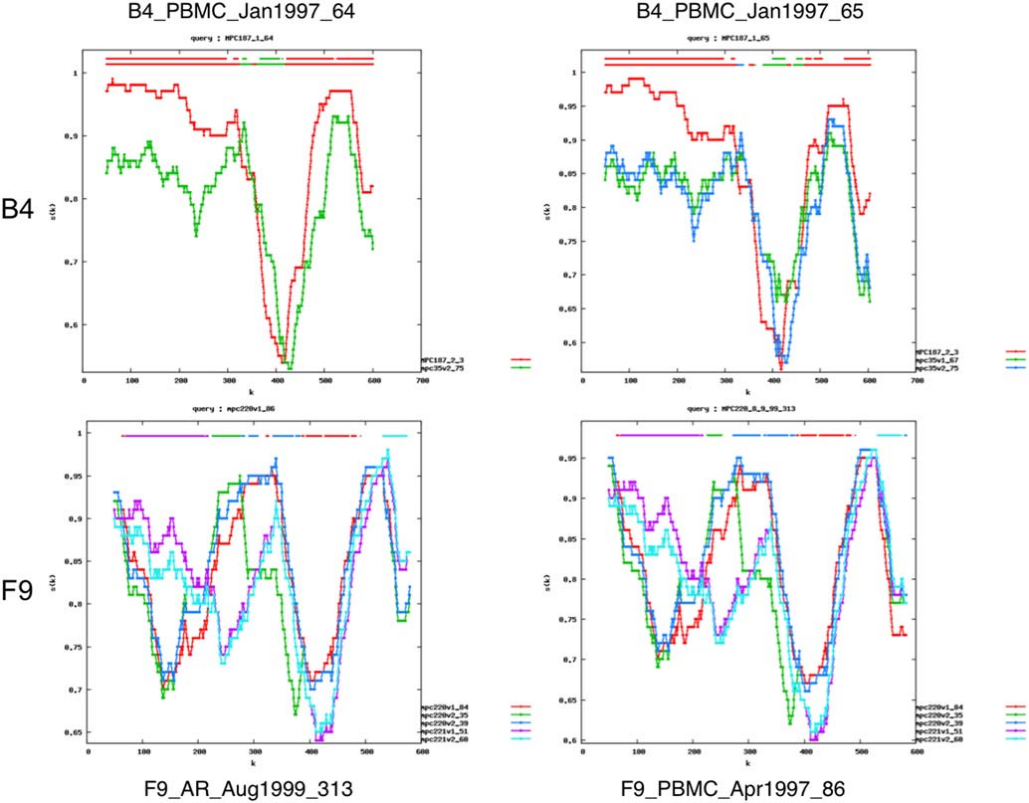
RIP 3.0 analysis. Subject 4 in Pair B had 2 sequences for which 2–3 segments were more similar to one of his partner's sequences than his own with a significance threshold of 90%, shown by parallel green horizontal lines above the curves. The areas of similarity were at positions 400–426 and 450–462 for the first sequence and 331–338, 365–407, and 412–414 in the second sequence. Subject 9 in Pair F had 2 sequences for which 3 segments were similar to his partner Subject 10, shown by purple and aqua lines above the curves. These areas of similarity did not meet the threshold for statistical significance of 90%. The areas of similarity were at positions 70–201, 204–207, and 519–552 in the first sequence and 70–211, 214–217, and 531–572 in the second sequence.

We analyzed sequences from pairs whose viruses were phylogenetically unlinked for evidence of subgenic linkage by assessment of viral recombination between sequences of each partner in the pair. Of the 5 pairs (A, B, E, F, and I), the algorithm RIP 3.0 identified potential recombinant sequences between partners in pairs B and F (Figure 4). Subject B4 had 2 PBMC-derived sequences from the first study visit that had regions similar to the sequences of his partner B3. In one sequence, there were 2 regions of similarity, 27 and 13 nucleotides in length, containing 15 and 7 shared informative sites that were detected by the InSites algorithm. In the other, there were 3 regions of similarity, 8, 42, and 3 nucleotides in length, containing 1, 32, and 3 shared informative sites. All of these regions met the cutoff for statistical significance at the 90% level. Subject F9 had 1 PBMC- and 1 anorectal-derived sequence similar to those of his partner F10, 1 from the first study visit and another 2 years post-enrollment. In one of these, there were 3 regions, 132, 4, and 44 nucleotides in length, containing 43, 2, and 5 shared informative sites, for which the closest matching sequence was from his partner F10. In the other sequence, there were also 3 regions of similarity, 142, 4, and 42 nucleotides in length, containing 45, 0, and 3 shared informative sites. However, none of the regions in either of F9's sequences met the significance threshold at the 90% confidence level.

**Figure 5:**
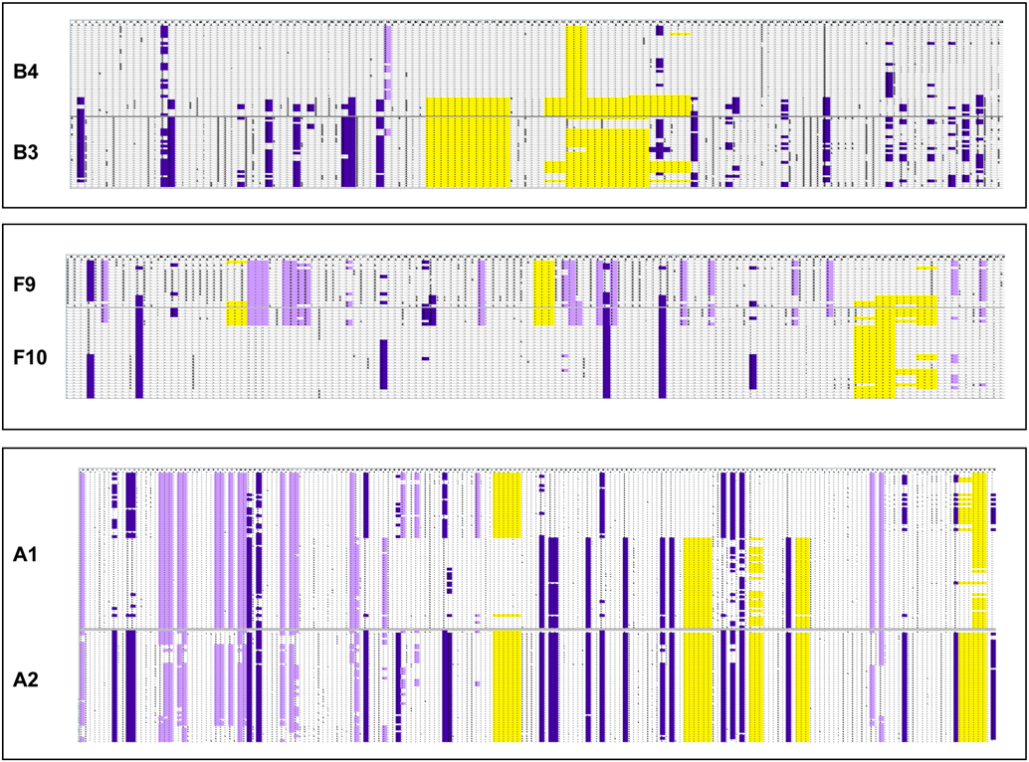
Alignments of phylogenetically informative sites for Pairs A, B, and F, with shading indicating shared regions. Lavender: nucleotide in upper partner's sequences shared with lower partner's sequences. Purple: nucleotide in lower partner's sequences shared with upper partner's sequences. Yellow: gap.

Restricting the alignment to phylogenetically informative sites using the InSites program, we were able to identify sites of genetic similarity between the partners in Pairs B and F (Figure 5). Pair B had 264 informative sites. There were 2 large gaps of 12 and 21 nucleotides present in a majority of B3's sequences, shared with 8 sequences from B4. These included 7 PBMC-derived sequences, 5 from the first and 2 from the final study visit, and 1 anorectal-derived sequence from the final study visit. In 15 other areas across the alignment that were 1–2 nucleotides in length, some or all of these 8 sequences from B4 resembled the majority of sequences from B3. Pair F had 203 informative sites. The 17 areas where 6 sequences from F10 resembled the majority of sequences from F9 were found across the entire alignment and ranged from 1–3 nucleotides in length. The 6 sequences from F10 included 4 anorectal-derived sequences from 3 time points after the first study visit and 1 PBMC-derived sequence from the final study visit. A gap region ranging from 3–9 nucleotides in length was also present in a majority of F10 and in 4 PBMC-derived sequences from F9, 1 from the first study visit and 3 from the final study visit.

In Pair A, for which RIP 3.0 did not identify recombinant sequences, InSites analysis did show areas of similarity. Of 245 total informative sites, 34 areas 1–2 nucleotides in length were present in a majority of one partner's sequences and shared by a subset of sequences in the other partner. In addition, there were 5 gaps 3–6 nucleotides in length present in nearly all of A2's sequences and a portion of A1's sequences. These sites were found across the alignment and involved both blood- and anorectal-derived viruses from all time points evaluated. No compelling evidence for genetic similarity suggestive of recombination was found in Pairs E and I.

We also compared InSites output with sequences from pairs of subjects in the cohort who were not expected to be epidemiologically linked to the results from the enrolled couples A, B, E, F, and I. The mean proportion of informative sites that was shared by at least 1 sequence from each individual was 30.4% (range 20.5–45.7%) in the enrolled couples versus 25.3% (range 15.7–37.3%) in 8 pairs without known epidemiologic linkage. However, there was no statistically significant difference in the means (t-test, 2-sided p = 0.33). The number of shared sites that neighbored one another did not appear to be different in the two groups, either.

## Discussion

In this study, we sought to determine whether or not chronically infected MSM on antiretroviral therapy can acquire HIV strains from their seroconcordant long-term sexual partners. Transmission may have occurred before the study period in 3 of the 8 enrolled couples, as the subjects had viruses that clustered monophyletically with those of their partners. In one of these 3 couples, both partners reported that they were HIV-infected before their relationship began. In this case, one subject may have superinfected the other, but we were unable to detect the strain he had acquired during primary infection. The other 5 couples had viruses that separated into unlinked polyphyletic lineages for each partner, with pairwise nucleotide distances consistent with independent sources of infection. In addition, no related viruses were found with strain-specific PCR in any of 3 evaluable pairs. Further investigation with recombination detection algorithms showed that viruses from one subject may have recombined with those from his partner in 2 of the 5 couples whose viruses were unlinked in the phylogenetic tree, but in only one of these cases was the threshold for statistical significance met. In summary, after intensive evaluation, using viral sequencing, phylogenetic analysis, strain-specific PCR, and recombination detection algorithms in conjunction with epidemiologic data, we found evidence suggestive of HIV superinfection in 2 of 8 HIV-1 seroconcordant MSM couples.

Although studies involving heterosexual couples, IDUs, and individuals under ART in the United States failed to detect HIV-1 superinfection, despite large numbers of subjects and long follow-up periods [41], [42], recent data from other cohorts indicate that dual and superinfection may be under-recognized. Three studies have detected dual and superinfection at unexpectedly high rates in ART-naïve individuals in Africa. Herbinger et al. reported the prevalence of dual infection to be 9 and 19%, respectively, in normal and high-risk cohorts in Tanzania [22] and Piantadosi et al. found superinfection incidence rates of 3.7% and 7.7% per year in two groups of female sex workers in Kenya [21], [23]. Analyses of viral recombination were used in all three of these studies. Moreover, additional data providing indirect support of sexual transmission of HIV-1 in seroconcordant MSM partners in the United States has emerged. Willberg et al. have demonstrated that men who did not have evidence of systemic superinfection, but whose seroconcordant partners were viremic, had increased HIV-specific T cell responses when compared to those whose partners had undetectable plasma viral loads [43]. These data suggest that occult superinfection in seroconcordant sexual partners may occur more commonly than previously appreciated.

Our study had several unique components. We had access to detailed sexual histories, confirming that risk behaviors were occurring. Second, the ART and STI histories for all individuals were available. Third, we utilized the additional laboratory method of strain-specific PCR, which could detect low levels of transmission with a higher level of sensitivity and specificity than typical PCR methods. Finally, because we had partner pairs, we were able to perform analysis for recombination between partners' strains in those pairs whose viruses appeared unrelated by tree- and distance-based phylogenetic methods and strain-specific PCR.

There are several potential causes for our inability to detect the parental strain of the superinfecting virus in couples B and D. First, we were unable to determine the precise timing of infection with the partner's virus, as this most likely occurred prior to enrollment in the study. During each successive cycle of viral replication, further recombination both obscures the ability to detect the parental strains and blurs recombination breakpoints. Second, all of these subjects received ART during at least some portion of the follow-up period. Although three individuals (D5, H13, and I15) had suppression of viral replication at all visits, none of these were in couples with transmission detected during the study period. It is reasonable to regard those who are taking antiretroviral medications as at least partially protected from acquisition of additional strains of HIV-1. Third, all viral sequences isolated from the cohort were of subtype B, whereas many of the reported dual and superinfection cases have occurred with viral strains of a subtype different than that of the subject's primary isolate [2]–[8], [21], [22]. The substantially greater divergence between viruses in inter-subtype transmissions facilitates the detection of superinfection and may account for their greater presence in the literature. However, it is also possible that adaptive immunity provides better protection against strains with a high degree of genetic similarity [17], [44]. Finally, all subjects in our study were in the chronic stage of HIV-1 infection, when the CD4^+^ T lymphocyte count almost invariably has decreased significantly from baseline, leaving fewer uninfected cells than in acute infection or after a period of successful antiretroviral therapy (reviewed in [45] and [46]). Therefore, in individuals with established HIV infection, fewer host cells are vulnerable to incoming new viral strains, which may reduce their risk for detectable superinfection.

The results we obtained from the two recombination detection algorithms were different. RIP, available for more than a decade from the Los Alamos National Laboratory and widely used, works by evaluating the similarity of a query sequence to an alignment while moving stepwise across the alignment. The InSites algorithm compares sequences in an alignment to a reference sequence and shows each phylogenetically informative site, defined as a site at which more than one sequence differs from the reference sequence. The potential benefit to using InSites is to locate shared sites in an alignment of sequences with limited diversity, where recombination breakpoints may be closer together than the minimum window size in a sliding window algorithm allows one to detect. While RIP 3.0 found 2 sequences in one individual that may have recombined with his partner's virus at a 90% confidence level, we were unable to detect a difference in the number of shared informative sites that InSites revealed in the enrolled couples versus the pairs with no known epidemiologic link. The small sample size in our cohort may explain our inability to detect a difference in shared informative sites between these 2 groups. However, it also may be the case that the overall number of shared sites is of lesser importance than their relative positions when evaluating for recombination.

Three of the couples in our study (D, G, and H) had viral sequences with a high degree of similarity (Figure 1). That initial acquisition of the same HIV strain could have occurred for pairs G and H can be inferred from their behavioral data in Table 1, showing that their partnership pre-dated their perceived year of HIV acquisition. However, the same conclusion cannot be drawn for couple D, as each reported acquiring HIV in 1988, 4 years before partnership formation. Given that separate phylogenetic trees from each of these pairs did not support ongoing transmission between partners during the study period, three explanations for the similarity between their sequences exist. First, one partner in the couple had transmitted HIV to the other before enrollment. Second, both partners were infected by a third source partner or other closely linked sources. Finally, HIV transmission from one partner to the other had occurred prior to study entry, with consequent overgrowth of the superinfecting strain, leading to inability to detect the primary infecting strain. The latter has been described in several case reports [4], [26]. Our study cannot formally distinguish among these possibilities.

In summary, in this cohort of 8 seroconcordant couples with chronic HIV-1 infection receiving ART, we observed one couple in which HIV superinfection may have occurred but was only detectable by recombination analysis and another couple in which one partner may have superinfected the other prior to study enrollment, based on epidemiologic and phylogenetic evidence. According to the objective clinical and self-reported behavioral data, the subjects in our cohort were at increased risk for sexually acquired superinfection. Their ART adherence was incomplete, as evidenced by the proportion of visits with detectable viral loads, a pattern not uncommon in ART-treated cohorts [47]. Although our study was not designed to determine whether the acquisition of additional viral strains led to clinical consequences, both dual and superinfection have been associated with antiretroviral drug resistance [24], [25] and accelerated progression to AIDS [10], [26]. The evidence for viral transmission in these couples should elicit concern on the part of HIV-infected persons with seroconcordant sexual partners. In particular, these data are a reminder that precautionary measures among HIV-infected individuals are necessary, as HIV-1 superinfection may occur even while under ART. Thus, we advocate that public health messages advising condom use [48]–[50] continue to be heeded, including by HIV-1 seroconcordant couples.
